# Circulating Tumor DNA Assay Detects Merkel Cell Carcinoma Recurrence, Disease Progression, and Minimal Residual Disease: Surveillance and Prognostic Implications

**DOI:** 10.1200/JCO.23.02054

**Published:** 2024-07-25

**Authors:** Tomoko Akaike, Manisha Thakuria, Ann W. Silk, Daniel S. Hippe, Song Youn Park, Naomi A. So, Nolan J. Maloney, Lindsay Gunnell, Alec Eschholz, Emily Y. Kim, Sumi Sinha, Evan Thomas Hall, Shailender Bhatia, Sunil Reddy, Angel Augusto Rodriguez, Alexey Aleshin, Jacob S. Choi, Kenneth Y. Tsai, Sue S. Yom, Siegrid S. Yu, Jaehyuk Choi, Sunandana Chandra, Paul Nghiem, Lisa C. Zaba

**Affiliations:** ^1^ University of Washington, Seattle, WA; ^2^ Brigham and Women's Hospital, Boston, MA; ^3^ Dana-Farber Cancer Institute, Boston, MA; ^4^ Harvard Medical School, Boston, MA; ^5^ Fred Hutchinson Cancer Center, Seattle, WA; ^6^ Stanford University School of Medicine, Palo Alto, CA; ^7^ University of California San Francisco, San Francisco, CA; ^8^ Natera, Inc, Austin, TX; ^9^ Northwestern University, Chicago, IL; ^10^ Moffitt Cancer Center, Tampa, FL

## Abstract

**PURPOSE:**

Merkel cell carcinoma (MCC) is an aggressive skin cancer with a 40% recurrence rate, lacking effective prognostic biomarkers and surveillance methods. This prospective, multicenter, observational study aimed to evaluate circulating tumor DNA (ctDNA) as a biomarker for detecting MCC recurrence.

**METHODS:**

Plasma samples, clinical data, and imaging results were collected from 319 patients. A tumor-informed ctDNA assay was used for analysis. Patients were divided into discovery (167 patients) and validation (152 patients) cohorts. Diagnostic performance, including sensitivity, specificity, positive predictive value (PPV), and negative predictive value (NPV), was assessed.

**RESULTS:**

ctDNA showed high sensitivity, 95% (discovery; 95% CI, 87 to 99) and 94% (validation; 95% CI, 85 to 98), for detecting disease at enrollment, with corresponding specificities of 90% (95% CI, 82 to 95) and 86% (95% CI, 77 to 93). A positive ctDNA during surveillance indicated increased recurrence risk, with hazard ratios (HRs) of 6.8 (discovery; 95% CI, 2.9 to 16) and 20 (validation; 95% CI, 8.3 to 50). The PPV for clinical recurrence at 1 year after a positive ctDNA test was 69% (discovery; 95% CI, 32 to 91) and 94% (validation; 95% CI, 71 to 100), respectively. The NPV at 135 days after a negative ctDNA test was 94% (discovery; 95% CI, 90 to 97) and 93% (validation; 95% CI, 89 to 97), respectively. Patients positive for ctDNA within 4 months after treatment had higher rates of recurrence, with 1-year rates of 74% versus 21% (adjusted HR, 7.4 [95% CI, 2.7 to 20]).

**CONCLUSION:**

ctDNA testing exhibited high prognostic accuracy in detecting MCC recurrence, suggesting its potential to reduce frequent surveillance imaging. ctDNA also identifies high-risk patients who need more frequent imaging and may be best suited for adjuvant therapy trials.

## INTRODUCTION

Merkel cell carcinoma (MCC) is a highly aggressive neuroendocrine skin cancer, associated with high mortality and a 40% recurrence rate within 5 years.^1^ To monitor for potential recurrence, patients are typically subjected to serial full-body imaging using computed tomography (CT) or positron emission tomography (PET)-CT for up to 5 years.^2,3^

**CONTEXT**

**Key Objective**
To evaluate whether tumor-informed circulating tumor DNA (ctDNA) is an accurate proxy for Merkel cell carcinoma (MCC) disease status and can predict recurrence.
**Knowledge Generated**
Among 319 patients with MCC stage I-IV across six sites, divided into discovery and validation cohorts, ctDNA has a sensitivity of 94%-95% and a specificity of 86%-90% for detecting clinically evident disease. In a subset analysis of stage I-III patients, the presence of a positive ctDNA within 4 months of completing curative treatment was associated with a significantly higher risk of recurrence (hazard ratio, 7.4), outperforming established MCC risk factors such as the presence of nodal disease, immunosuppression, sex, and age.
**Relevance *(R.G. Maki)***
ctDNA represents an important biomarker associated with recurrence risk in MCC. It should provide a means to intervene in order to improve patient outcomes with this unique form of skin cancer.*
*Relevance section written by *JCO* Associate Editor Robert G. Maki, MD, PhD, FACP, FASCO.

As the majority of MCC cases are caused by clonal integration of the Merkel cell polyomavirus (MCPyV) into the tumor genome, the MCPyV oncoprotein antibody test is a serology assay that has been used as a tumor marker.^4^ However, only approximately 50% of patients produce MCPyV oncoprotein antibody at the time of disease presence, antibody titers fall slowly over months after the disease is removed, and titers are less reliable after the first recurrence.^5,6^ Therefore, there is a need for an effective, blood-based biomarker of MCC disease that can be used to stratify patients with high-risk MCC, regardless of viral status.

Circulating tumor DNA (ctDNA) is a minimally invasive biomarker that measures cell-free DNA fragments in plasma.^7^ In recent years, there has been increasing evidence demonstrating the prognostic and predictive value of tumor-informed ctDNA assays in solid tumors such as melanoma, lung, bladder, and colorectal cancers for identifying molecular residual disease, early detection of recurrence, as well as monitoring response to systemic treatment.^8-12^ In melanoma, tumor-informed ctDNA was recently demonstrated to be more sensitive and specific (83% and 96%, respectively) than previously explored ctDNA methodologies such as digital droplet polymerase chain reaction, which detects single hotspot mutations in BRAF, NRAS, KIT, or TERT promoter, which had sensitivities ranging from 20% to 62%.^8,13-15^ In MCC, recent case series suggested that ctDNA could be a sensitive and predictive biomarker; however, the cohort sizes were too small to draw strong conclusions for clinical practice.^16-18^

In this prospective, multicenter, observational study, we assessed the utility of tumor-informed ctDNA for the detection of disease in patients with MCC. We evaluated ctDNA levels at the time of presentation and after initial treatment to determine whether ctDNA could detect clinically evident disease and identify high-risk patients who are likely to have a recurrence.

## METHODS

### Study Design and Population

**FIG A1.**
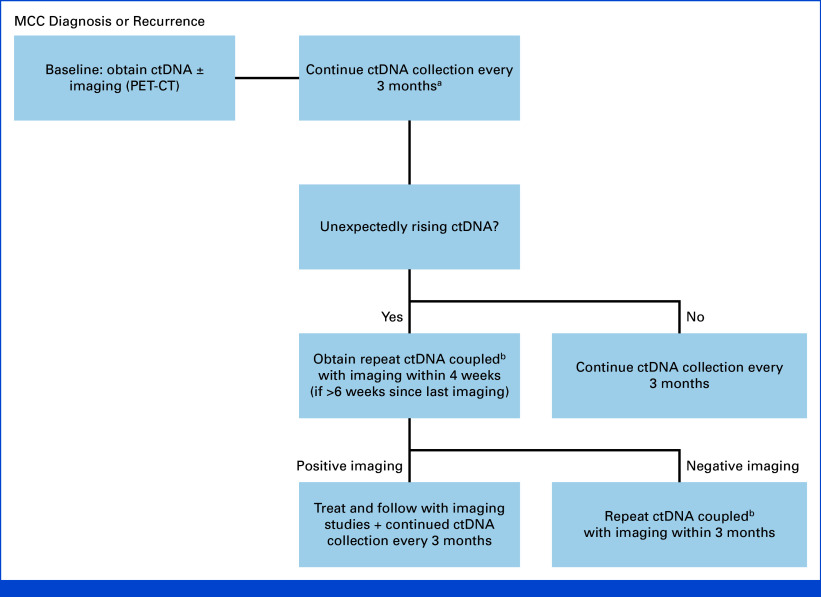
Protocol flowchart for ctDNA testing and imaging. Ordering of ctDNA testing and imaging was guided by the flowchart at all sites in the discovery and validation cohorts throughout the study period. CT, computed tomography; ctDNA, circulating tumor DNA; MCC, Merkel cell carcinoma; PET, positron emission tomography. ^a^For every patient, including after a recurrence is noted. ^b^Coupled is defined by ctDNA collection and imaging ordered within 4 weeks of each other.

This study was designed as a prospective, multicenter, and observational study of stage I-IV patients with histologically confirmed MCC with a discovery cohort and a validation cohort. All patients provided written informed consent approved by the institutional review board (IRB) at each participating center, and a data-sharing agreement was procured between the institutions. Patients were enrolled between April 2020 and August 2022, and the data cutoff dates were July 8, 2022, and August 31, 2022, for discovery and validation cohorts, respectively. Patients were eligible for enrollment at any time during their disease course, including before or after treatment. Blood samples were collected for ctDNA testing at the time of enrollment, and every 3 months during the surveillance period. Imaging studies, including CT, PET-CT, magnetic resonance imaging, or ultrasound, were obtained at enrollment for primary tumors and per National Comprehensive Cancer Network guidelines for patients in surveillance. If there was an unexpected rise in ctDNA, an additional ctDNA test was performed coupled with an imaging study within 4 weeks (protocol flowchart shown in Appendix Fig A1, online only). Clinical details, including follow-up, disease status at the time of enrollment, recurrences, and imaging results, were collected. Clinically evident disease was defined as MCC that was detected through physical examination, imaging studies, or tissue biopsy. Patients with incomplete clinical data, unattainable ctDNA assay development because of insufficient tissue, or sequencing failure because of comorbid hematologic malignancy or transplant were excluded. Inclusion and exclusion criteria for subgroup analyses are described in each objective in the section below.

### ctDNA Assay Using Multiplex Polymerase Chain Reaction–Based Next-Generation Sequencing

Tumor-informed ctDNA assays were designed for each patient as previously described.^11,19^ The ctDNA assay was centralized and conducted by Natera Inc, the developer and provider of the Signatera assay. Briefly, up to 16 patient-specific, somatic single-nucleotide variants (SNVs) were selected by performing whole-exome sequencing on formalin-fixed paraffin-embedded tumor tissue and matched normal blood samples. The multiplex polymerase chain reaction primers targeting the selected SNVs were used to detect ctDNA in patients' plasma samples. As previously described, plasma samples with ≥two SNVs detected were reported to the clinician in a standardized report as having a mean tumor molecule (MTM) per mL of plasma as >0.00.^9-11,20-24^

### Discovery and Validation Cohorts

Patients enrolled by Stanford University and University of Washington were designated as the discovery cohort and patients enrolled by Dana-Farber Cancer Institute, Northwestern University, University of California San Francisco, and Moffitt Cancer Center were designated as the validation cohort. Enrollment and data collection ran from April 2020 through August 2022 in the discovery cohort and from February 2021 through July 2022 in the validation cohort.

**FIG 1.**
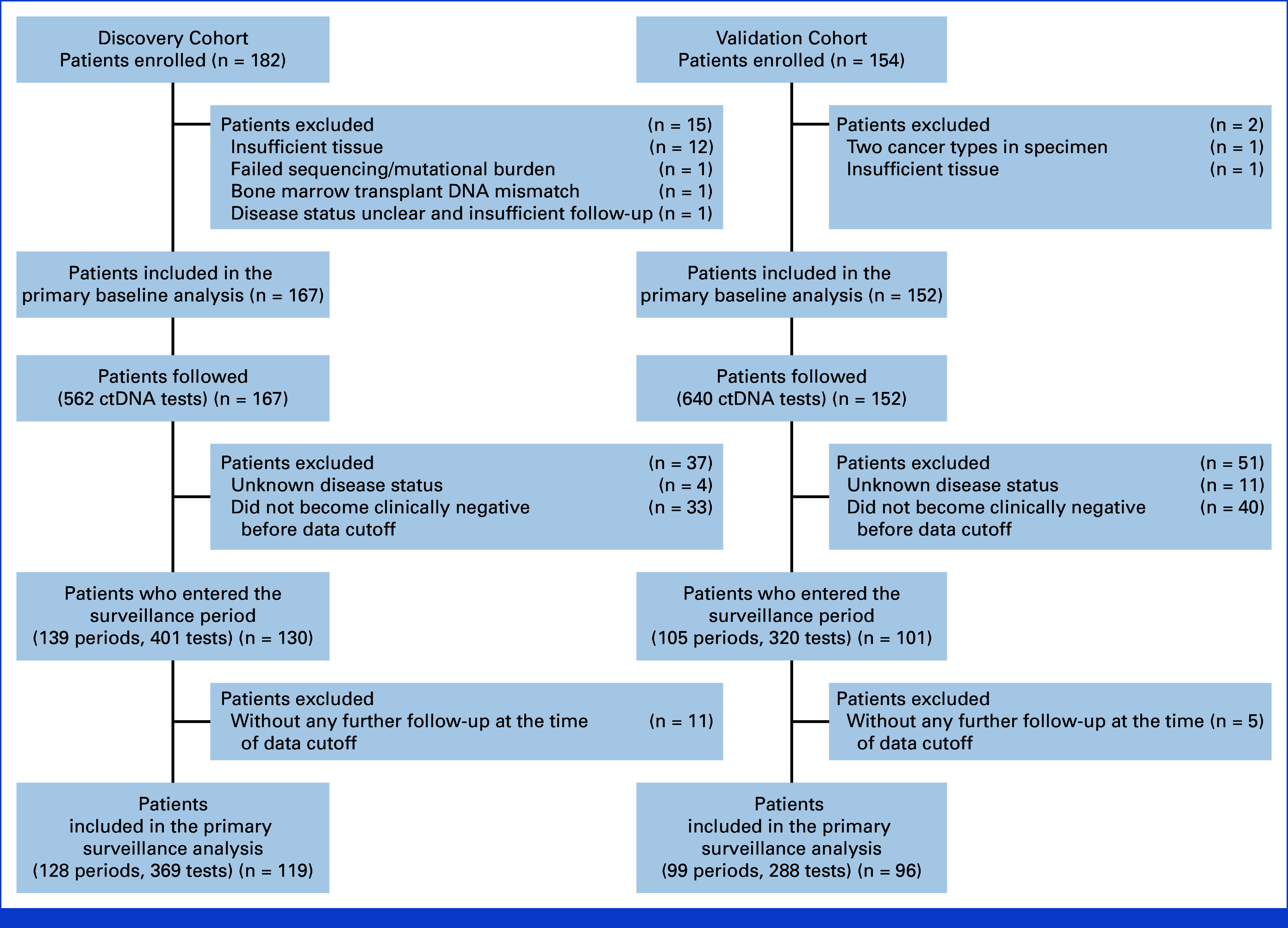
Flow diagram for patients with MCC enrolled and analyzed in the discovery and validation cohorts. Patients enrolled at University of Washington and Stanford University were designated the discovery cohort. Patients enrolled at Dana-Farber Cancer Institute, Northwestern Memorial Hospital, University of California San Francisco, and Moffitt Cancer Center were designated the validation cohort. ctDNA, circulating tumor DNA; MCC, Merkel cell carcinoma.

**FIG A2.**
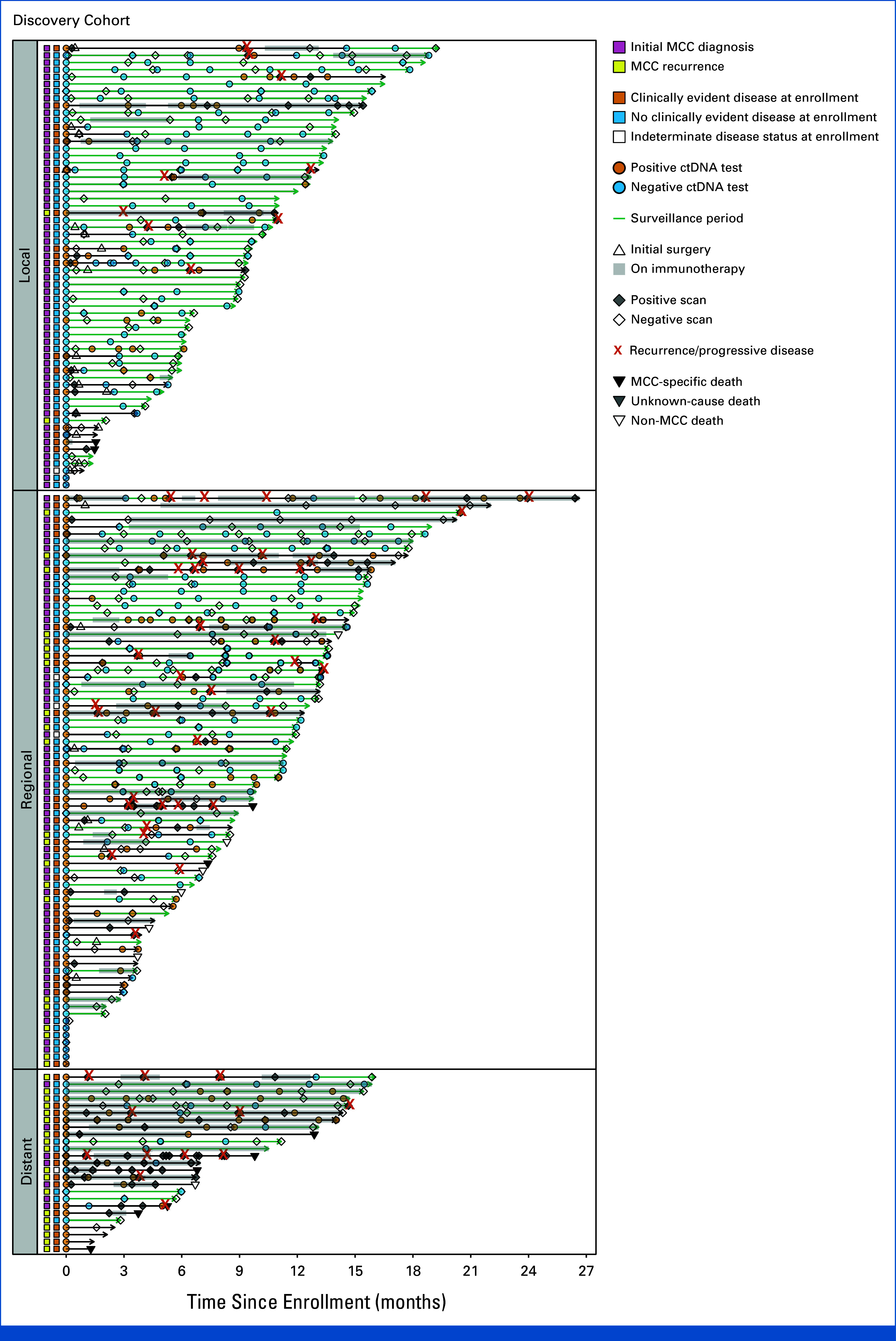
Swimmer plot of patients in the discovery cohort, categorized by local MCC, regional disease, or distant MCC at the time of enrollment. All 167 patients included in the discovery cohort are depicted, starting from the time of enrollment. Categorization into local, regional, and distant MCC was based on AJCC eighth edition staging at the initial MCC diagnosis or most recent recurrence. All ctDNA tests performed during each patient's study follow-up are shown using orange circles (positive test) and blue circles (negative test). Surveillance periods, between when a patient was determined clinically to be negative for disease and a recurrence or end of follow-up, are indicated by the green lines. Recurrences or disease progression (X), death (upside-down triangle), and other relevant treatments and procedures are also depicted. AJCC, American Joint Committee on Cancer; ctDNA, circulating tumor DNA; MCC, Merkel cell carcinoma.

**FIG A3.**
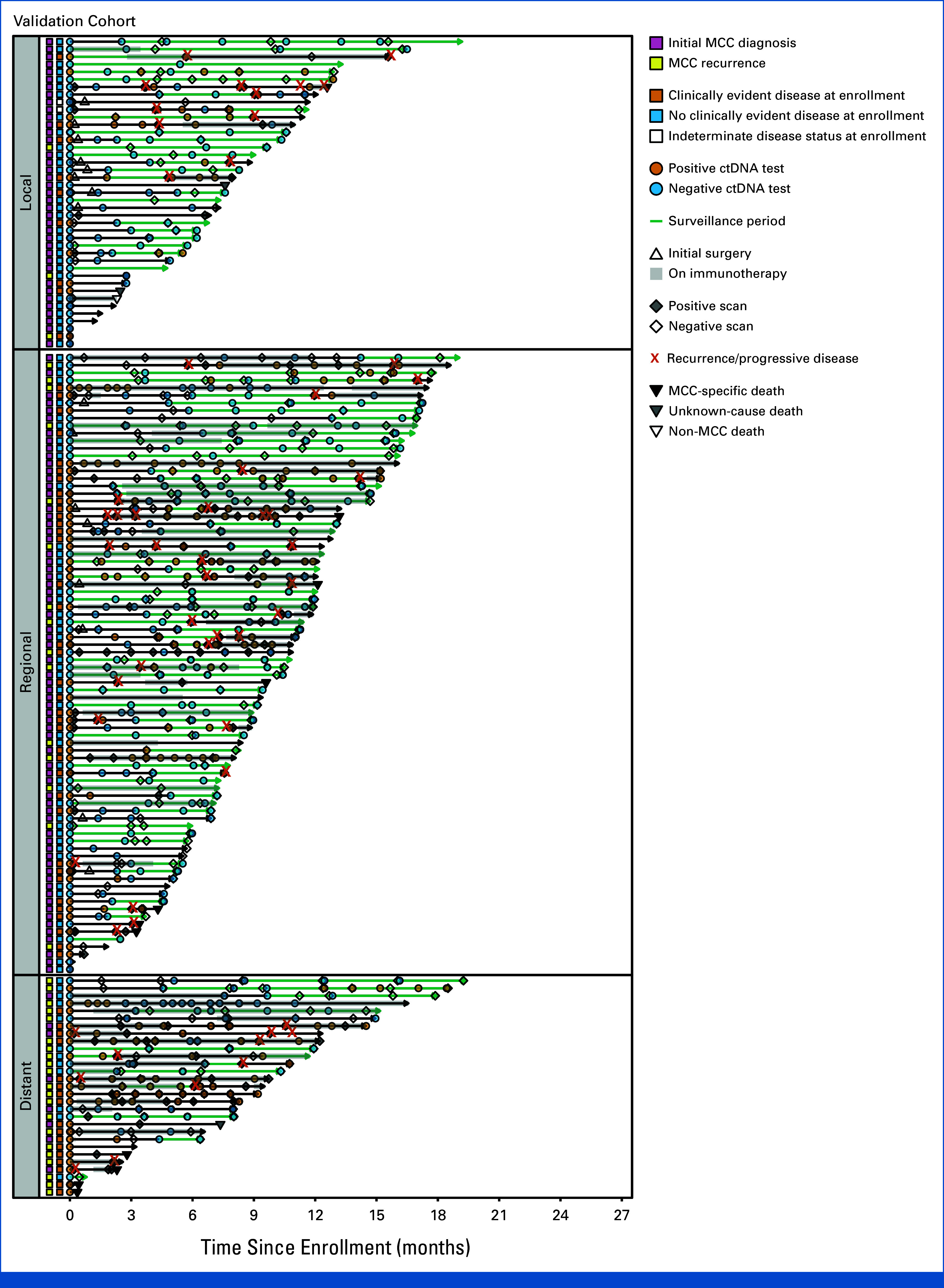
Swimmer plot of patients in the validation cohort, categorized by local MCC, regional disease, or distant MCC at the time of enrollment. All 152 patients included in the validation cohort are depicted, starting from the time of enrollment. Categorization into local, regional, and distant MCC was based on AJCC eighth edition staging at the initial MCC diagnosis or most recent recurrence. All ctDNA tests performed during each patient's study follow-up are shown using orange circles (positive test) and blue circles (negative test). Surveillance periods, between when a patient was determined clinically to be negative for disease and a recurrence or end of follow-up, are indicated by the green lines. Recurrences or disease progression (X), death (upside-down triangle), and other relevant treatments and procedures are also depicted. AJCC, American Joint Committee on Cancer; ctDNA, circulating tumor DNA; MCC, Merkel cell carcinoma.

A total of 336 patients were initially enrolled across six study sites. Among them, 17 patients were excluded as shown in Figure 1. The remaining 319 patients were included in the analysis, with 167 in the discovery cohort and 152 in the validation cohort. Patient characteristics for the discovery and validation cohorts are summarized in Appendix Table A1 and the Data Supplement (Methods, online only). A total of 562 ctDNA tests were performed over a median follow-up of 295 days in the discovery cohort (median [IQR], 3 [2-5] tests per patient) and 640 ctDNA tests were performed over a median follow-up of 284 days in the validation cohort (median [IQR], 3 [2-4] tests per patient). Swimmer plots of all patients in the discovery cohort (Appendix Fig A2) and the validation cohort (Appendix Fig A3) showing individual-level data on stage, disease status, treatments, ctDNA tests, scans, and outcomes over time are included in the Data Supplement.

### End Points and Assessments

Primary analyses included (1) ctDNA test sensitivity and specificity for disease status at enrollment; (2) risk of recurrence stratified by ctDNA status on the basis of serial ctDNA testing during surveillance; and (3) positive predictive value (PPV) and negative predictive value (NPV) of the ctDNA test for predicting clinical recurrence at each time point during surveillance. Secondary analyses included (1) correlating quantitative ctDNA level with primary tumor size; (2) quantifying risk of recurrence at varying levels of ctDNA positivity, and (3) evaluating whether the detection of ctDNA after completing initial treatment can predict recurrence and risk stratify patients.

### Statistical Analysis

The Reporting Recommendations for Tumor Marker Prognostic Studies guidelines were followed in the analysis and reporting of results (Appendix Table A2).^25^ A detailed statistical analysis plan is included in the Data Supplement. All *P* values were two-sided and statistical significance was defined as *P* < .05 without adjustment for multiple testing.

The primary analysis plan was developed using the discovery cohort before any statistical analysis of the validation cohort. The study protocol and procedures were not altered over the study period on the basis of any data analysis results. Primary end point analyses were performed on each cohort separately. These primary analyses were performed first on the discovery cohort while developing the analysis plan and determining the ctDNA MTM/mL threshold for detection of MCC, and second on the validation cohort as a prespecified analysis to achieve unbiased estimates of ctDNA performance for the detection of MCC. Secondary end point analyses were conducted using both the discovery and validation cohorts combined as a single cohort to maximize the available sample size. The division of end points into primary and secondary was done on the basis of the results from the discovery cohort, before any analysis of the validation cohort.

### Primary End Point Analyses

The sensitivity and specificity of the ctDNA test for detecting clinically evident disease at enrollment were estimated using the first ctDNA test for all patients with determinable clinical disease status. The performance of the ctDNA test at different thresholds was also considered using receiver operating characteristic (ROC) curve analysis and summarized using the AUC. The ROC analysis of ctDNA in the discovery cohort was used to determine the ctDNA threshold for positivity to be assessed in the validation cohort. In the surveillance setting, the association of ctDNA status with risk of recurrence during serial testing was performed by stratifying patients as ctDNA-positive at any time point versus patients who remained ctDNA-negative, where ctDNA status was treated as a time-varying covariate to account for immortal time bias.^26^ Cox regression models were used to compare recurrence risk between positive and negative groups while adjusting for other risk factors. The PPV and NPV of ctDNA status for recurrence during surveillance were estimated at multiple intervals after a positive or negative test using the cumulative incidence function estimator. The unit of analysis for PPV and NPV was the ctDNA test, and the time to recurrence after each test was defined as the time from that test to clinical detection of recurrence, censored at the last follow-up. Clustered bootstrapping was used to account for the nonindependence of multiple tests per patient.^27^

### Secondary End Point Analyses

Statistical methods for the secondary end point analyses are provided in the Data Supplement.

### IRB

All studies were performed in accordance with the Declaration of Helsinki and were approved by the IRB (protocol code Stanford IRB 61461, University of Washington/Fred Hutch Cancer IRB 6585, Dana-Farber/Harvard Cancer Center IRB 09-156, Northwestern University IRB STU00216228, University of California San Francisco IRB 21-35252, and Moffitt Cancer Center IRB 00000971). All patients included in this study provided informed consent for their clinical data to be analyzed for research purposes.

## RESULTS

### Correlation of ctDNA Test Results With Clinical Disease at Enrollment

**FIG 2.**
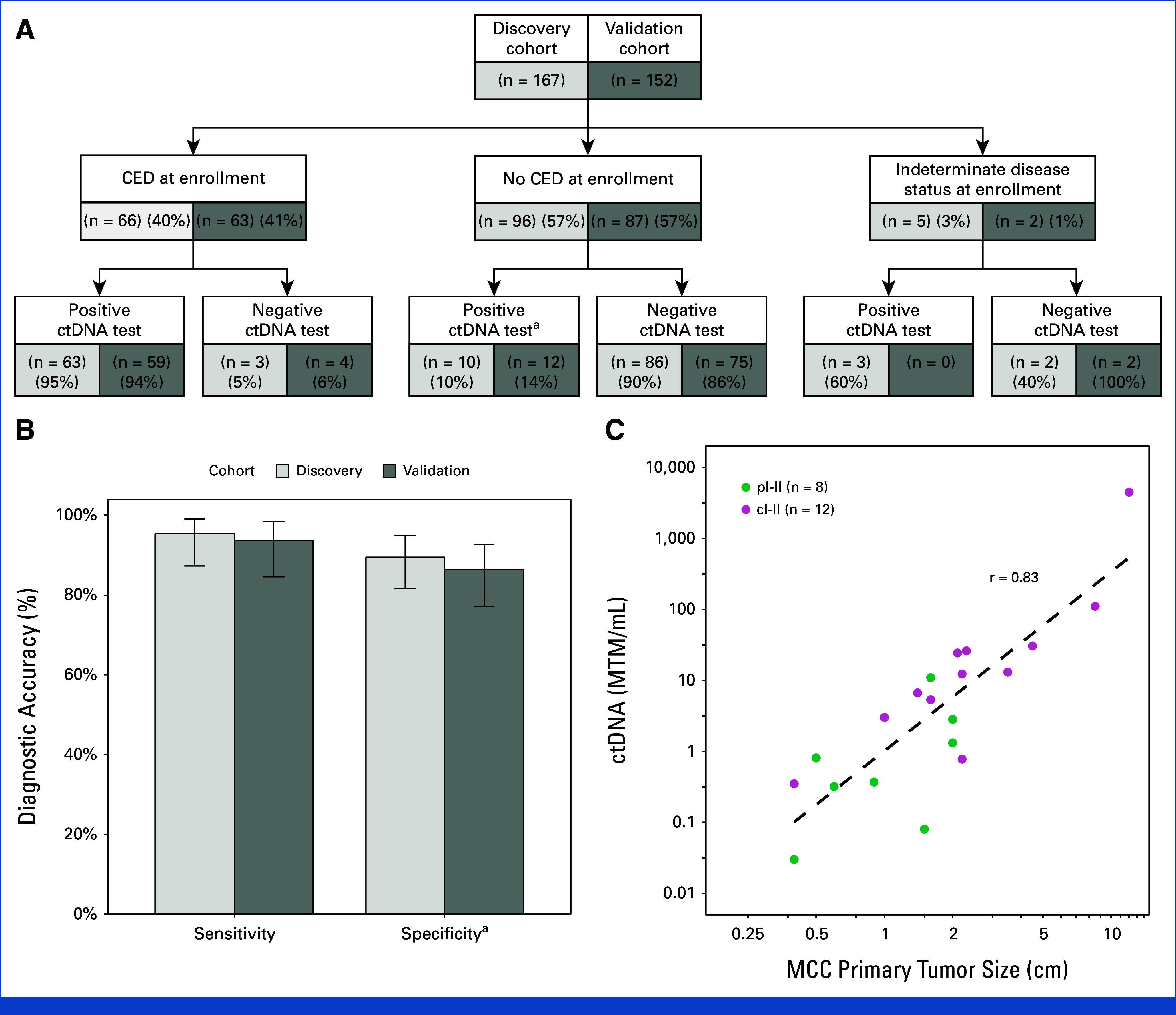
Diagnostic performance of ctDNA at enrollment for MCC disease status in the discovery and validation cohorts. (A) Flowchart for sensitivity and specificity calculations on the basis of disease status and ctDNA status at enrollment. (B) Diagnostic accuracy of ctDNA at enrollment. The sensitivity of ctDNA for CED, defined as detection of MCC on imaging or physical examination, at enrollment was 63/66 (95%; 95% CI, 87 to 99) in the discovery cohort and 59/63 (94%; 95% CI, 84 to 99) in the validation cohort. The specificity of ctDNA at enrollment was 86/96 (90%; 95% CI, 82 to 95) in the discovery cohort and 75/87 (86%; 95% CI, 77 to 93) in the validation cohort. (C) Relationship of primary tumor size (median, 1.8 cm; IQR, 1.0-2.2 cm; range, 0.4-12 cm) and the corresponding ctDNA level at enrollment (median, 4.2 [MTM/mL; IQR, 0.7-16 MTM/mL; range, 0.03-4490 MTM/mL) in stage I-II patients with detectable ctDNA, local disease only, and enrolled before initial treatment (n = 20; Spearman's *r* = 0.83; *P* < .001). CED, clinically evident disease; ctDNA, circulating tumor DNA; MCC, Merkel cell carcinoma; MTM, mean tumor molecule. ^a^Specificity was based on the absence of clinically evident disease at the time of enrollment, without consideration of subsequent recurrences.

In the discovery cohort at enrollment, 40% (66/167) of patients had clinically evident disease. Of these 66 patients, ctDNA positivity was detected in 63 patients, yielding a sensitivity of 95% (95% CI, 87 to 99; Figs 2A and 2B). Likewise, for specificity, 96 patients showed no clinically evident disease, and of these, 86 tested ctDNA-negative, yielding a specificity of 90% (95% CI, 82 to 95).

**FIG A4.**
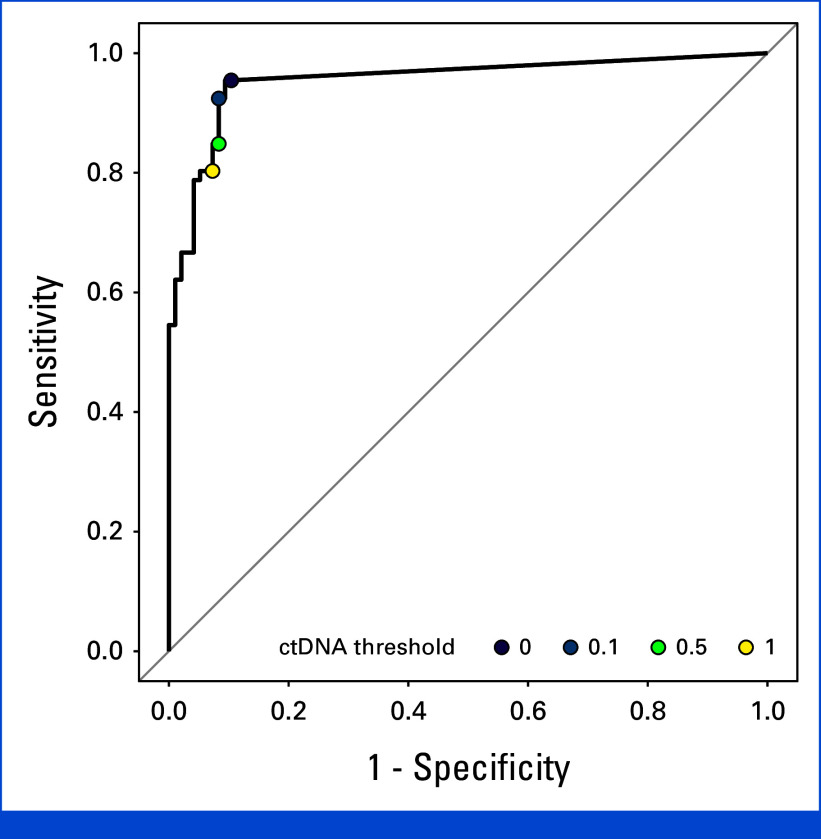
ROC curve for performance of ctDNA at enrollment for Merkel cell carcinoma disease status in the discovery cohort. The AUC of ctDNA in the discovery cohort was 0.95 (95% CI, 0.92 to 0.99). Sensitivity decreased rapidly as the ctDNA threshold was increased from 0.00 MTM/mL with minimal improvement specificity. To avoid this disproportionate loss of sensitivity, the ctDNA threshold for positivity of ctDNA >0.00 MTM/mL was used for validation in the validation cohort. ctDNA, circulating tumor DNA; MTM, mean tumor molecule; ROC, receiver operating characteristic.

Higher thresholds for ctDNA positivity were also considered using ROC analysis (Appendix Fig A4). Overall, the ctDNA level at enrollment had an AUC of 0.95 (95% CI, 0.92 to 0.99) for discriminating between clinically evident disease and no clinically evident disease in the discovery cohort. Sensitivity dropped rapidly as the ctDNA threshold was increased from 0.00 MTM/mL with minimal improvement in specificity (ctDNA >1 MTM/mL had a sensitivity and a specificity of 80% [53/66] and 93% [89/96], respectively). To avoid this disproportionate loss of sensitivity, the threshold for ctDNA positivity of ctDNA >0.00 MTM/mL was used for validation in the validation cohort.

In the validation cohort at enrollment, 41% (63/152) of patients had clinically evident disease. The sensitivity and specificity of ctDNA positivity (ctDNA >0.00 MTM/mL) were 94% (59/63; 95% CI, 85 to 98) and 86% (75/87; 95% CI, 77 to 93), respectively, similar to the performance in the discovery cohort (Figs 2A and 2B).

In the combined cohort, we also explored whether diagnostic performance differed by whether the patient was on immunotherapy at enrollment (n = 48) or not (n = 264). Both sensitivity (95% *v* 94%; *P* > .99) and specificity (81% *v* 89%; *P* = .33) were similar in patients on immunotherapy versus not on immunotherapy (Appendix Table A3). Additionally, ctDNA level and primary tumor diameter were significantly correlated (Spearman's *r* = 0.83; *P* < .001; Fig 2C) among the 20 patients enrolled before initial treatment with local disease only and detectable ctDNA.

### Risk of Recurrence Stratified by ctDNA Status During Surveillance

**FIG 3.**
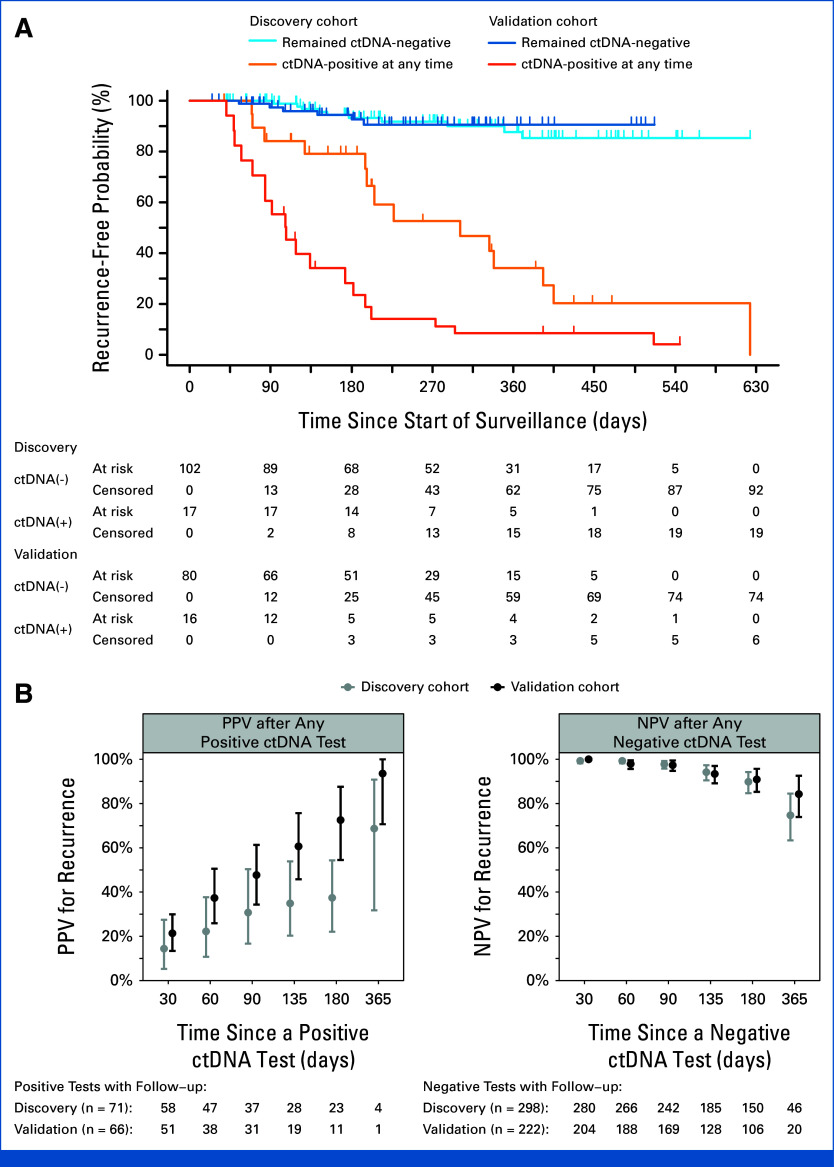
Risk of recurrence by ctDNA status and diagnostic accuracy of ctDNA during the surveillance period. (A) Recurrence-free probability stratified by ctDNA status. The recurrence-free probability after a positive ctDNA test (orange) at any point during disease course was significantly lower than when ctDNA tests were persistently negative (blue) in both the discovery cohort (dashed curves; HR, 6.8 [95% CI, 2.9 to 16]; *P* < .001) and the validation cohort (solid curves; HR, 20 [95% CI, 8.3 to 50]; *P* < .001). (B) PPV and NPV for subsequently detected recurrence over different time frames after each ctDNA test. Error bars indicate 95% CIs. NPV remained high at 135 days (4.5 months) after each negative ctDNA test in the discovery cohort (gray points; NPV, 94%; 95% CI, 90 to 97) and the validation cohort (black points; NPV, 93%; 95% CI, 89 to 97). ctDNA, circulating tumor DNA; HR, hazard ratio; NPV, negative predictive value; PPV, positive predictive value.

**FIG A5.**
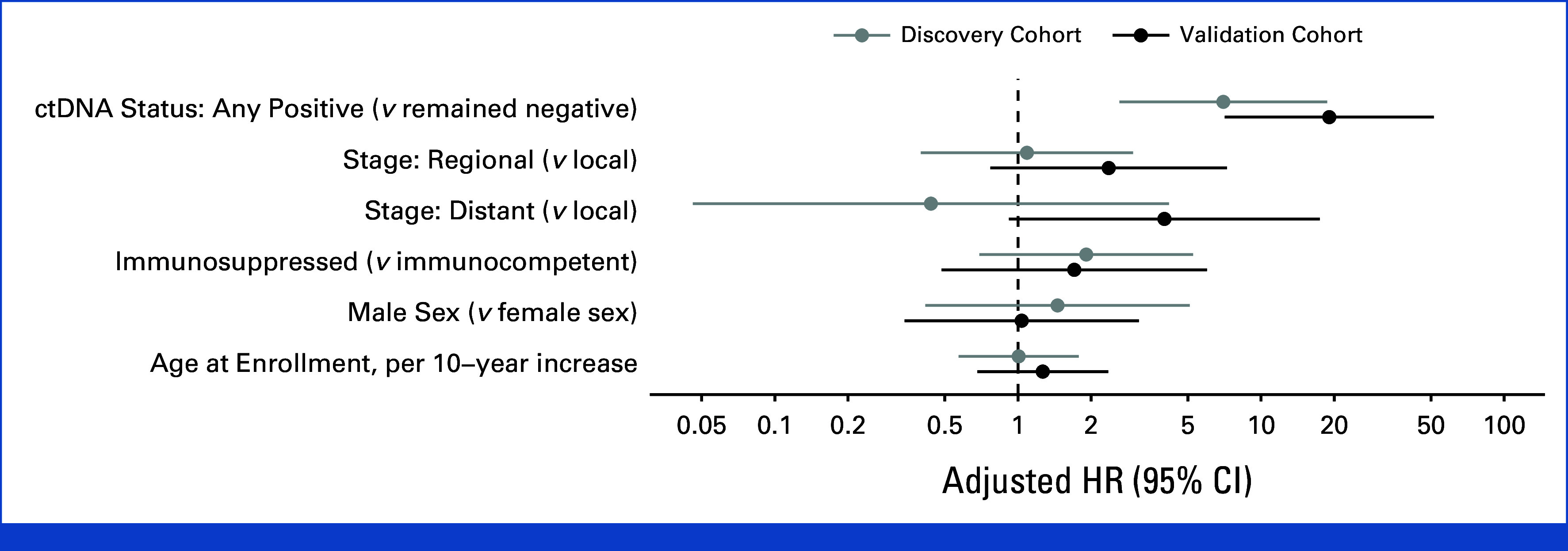
HRs relating ctDNA status and other risk factors with recurrence during surveillance. HRs are adjusted for all other factors shown. Patients who were ctDNA-positive at any point during surveillance compared with those who remained ctDNA-negative in both the discovery cohort (HR, 7.0 [95% CI, 2.6 to 18.7]; P < .001) and the validation cohort (HR, 19 [95% CI, 7.1 to 51]; P < .001) after accounting for other risk factors. Detailed numeric results are shown in Appendix Table A4. CI, confidence interval; ctDNA, circulating tumor DNA; HR, hazard ratio.

During surveillance, 119 patients (369 plasma samples) from the discovery cohort and 96 patients (288 plasma samples) from the validation cohort underwent serial ctDNA testing (Fig 1). These were stage I-IV patients who clinically had no evidence of disease at the start of surveillance. The median interval between ctDNA tests was 91 days (IQR, 77-107) in the discovery cohort and 83 days (IQR, 56-98) in the validation cohort. Among these, 24 patients in the discovery cohort recurred and two died over a median follow-up of 267 days. Comparatively, 25 patients in the validation cohort recurred with no deaths over a median follow-up of 194 days. The risk of recurrence was significantly higher in patients who were ctDNA-positive at any point during surveillance compared with those who remained ctDNA-negative in both the discovery cohort (hazard ratio [HR], 6.8 [95% CI, 2.9 to 16]; *P* < .001) and the validation cohort (HR, 20 [95% CI, 8.3 to 50]; *P* < .001; Fig 3A). These differences in recurrence between ctDNA-positive and ctDNA-negative groups remained significant after adjusting for stage, immunosuppression status, sex, and age in both the discovery cohort (*P* < .001) and the validation cohort (*P* < .001; Appendix Tables A4 and A5 and Fig A5).

### Performance of Serial ctDNA Testing for Predicting Clinical Recurrence During Surveillance

The cumulative incidence of clinical recurrence after each ctDNA test during surveillance, stratified by positive and negative ctDNA status, was used to calculate PPV and NPV. A gradual increase in PPV was observed for both discovery and validation cohorts at various time points, yielding a PPV of 69% (95% CI, 32 to 91) and 94% (95% CI, 71 to 100) at the 1-year time point, respectively (Fig 3B). NPV was observed to be high at 135 days (4.5 months) after any negative ctDNA test in both cohorts at 94% (95% CI, 90 to 97) and 93% (95% CI, 89 to 97; Fig 3C) and remained comparatively high at 180 days with 90% (95% CI, 85 to 94) and 91% (95% CI, 85 to 96) for the discovery and validation cohorts, respectively. The time between positive and negative ctDNA tests and subsequent imaging is summarized in Appendix Table A6.

### Relationships of Quantitative ctDNA Level and Likelihood of Clinical Recurrence Detection During Surveillance

**FIG 4.**
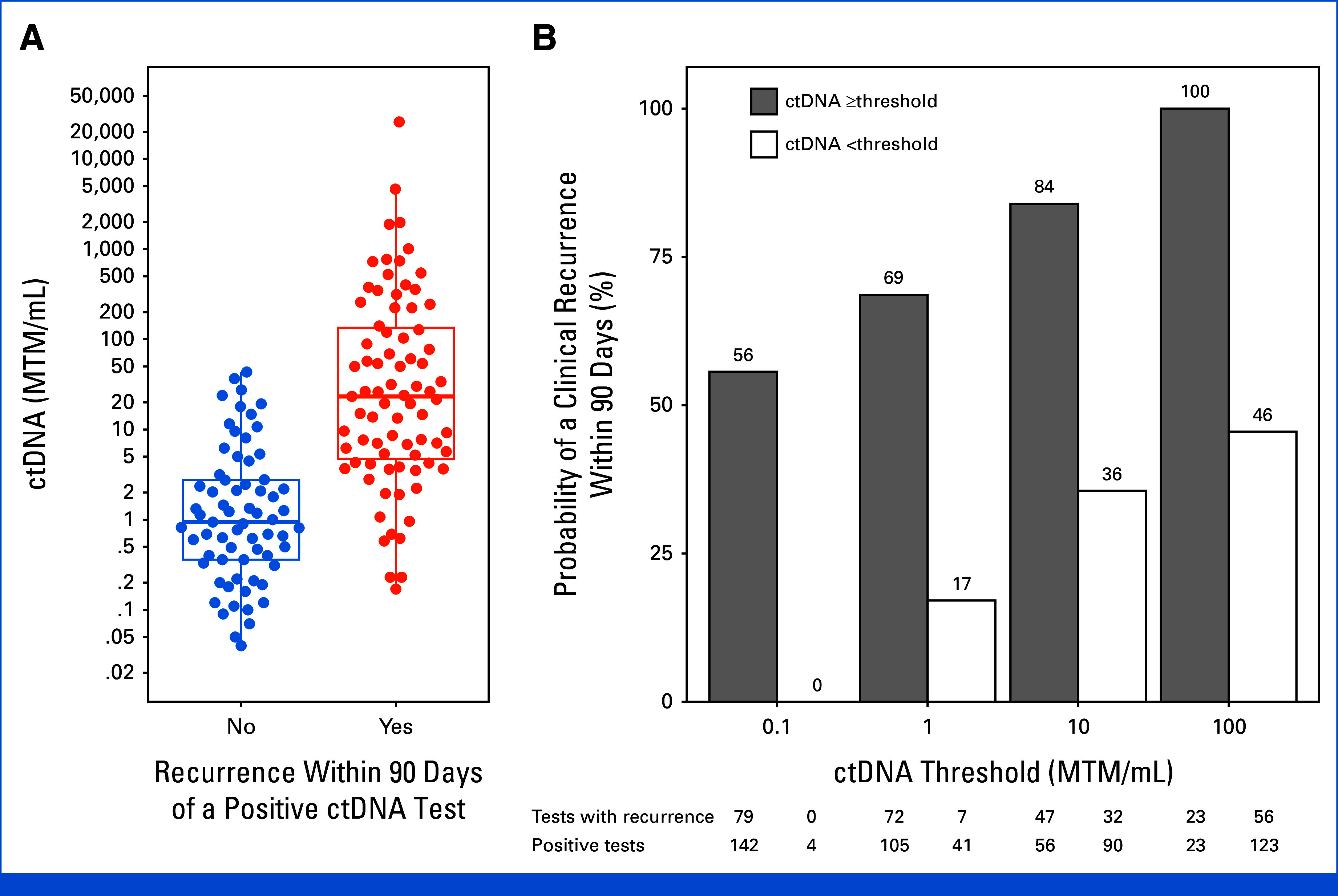
Likelihood of clinical detection of recurrence at different quantitative levels of ctDNA. (A) ctDNA levels from positive tests, stratified by whether the positive test was within 90 days of a clinical recurrence. Units are MTM per mL. Positive ctDNA levels drawn within 90 days of a recurrence (n = 79 tests) were significantly higher than levels drawn >90 days before a recurrence (n = 67 tests; *P* < .001). (B) Estimated likelihood of a recurrence being clinically detectable within 90 days before or after a positive ctDNA test, stratified at different ctDNA levels. Gray bars show risk of clinical recurrence when the ctDNA level was at or higher than the given threshold on the *x*-axis and the white bars show the risk of clinical recurrence when the ctDNA level was positive but below the given threshold. ctDNA, circulating tumor DNA; MTM, mean tumor molecule.

**FIG A6.**
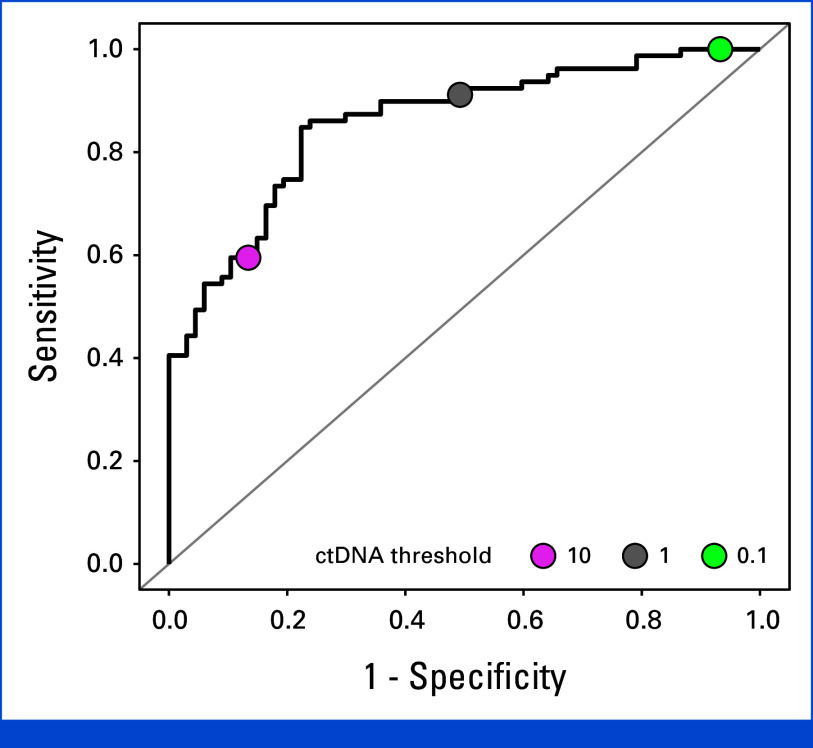
ROC curve for recurrence within 90 days of a positive test on the basis of quantitative ctDNA levels. All positive ctDNA tests from the surveillance period with at least 90 days of follow-up after the test or within 90 days of a recurrence, before or after, were included (n = 146 tests). Quantitative ctDNA levels were compared between tests drawn within 90 days of a recurrence (n = 79 tests) and tests drawn >90 days before a recurrence (n = 67 tests). The area under the curve was 0.86 (95% CI, 0.80 to 0.92). Three ctDNA thresholds (10 MTM/mL, 1 MTM/mL, and 0.1 MTM/mL) are marked on the curve (magenta, gray, and green points, respectively) to illustrate the sensitivity and specificity of different cutpoints. ctDNA, circulating tumor DNA; MTM, mean tumor molecule; ROC, receiver operating characteristic.

We next investigated the association of ctDNA levels (MTM/mL) with the percentage of patients experiencing recurrence in both cohorts combined. During surveillance, a total of 146 positive ctDNA tests from 61 patients had at least 90 days of follow-up after the positive test or were within 90 days before or after a clinical recurrence. The quantitative ctDNA levels were significantly higher among the 79 positive tests in which a recurrence was noted within 90 days before or after the positive test compared with the 67 positive tests where no recurrence was noted (median [IQR], 23 [5-134] *v* 0.9 [0.4-2.8] MTM/mL; *P* < .001; Fig 4A). The corresponding AUC was 0.86 (95% CI, 0.80 to 0.92; Appendix Fig A6). Among these 146 positive tests, the estimated risk of clinical recurrence detection within 90 days was 69% (72/105) when ctDNA was above 1 MTM/mL and 17% (7/41) when ctDNA was below 1 MTM/mL. The estimated risk was 100% (23/23) when ctDNA was above 100 MTM/mL and 46% (56/123) when ctDNA was positive and below 100 MTM/mL (Fig 4B).

### Prognosis Stratified by Post-Treatment ctDNA Status

**FIG 5.**
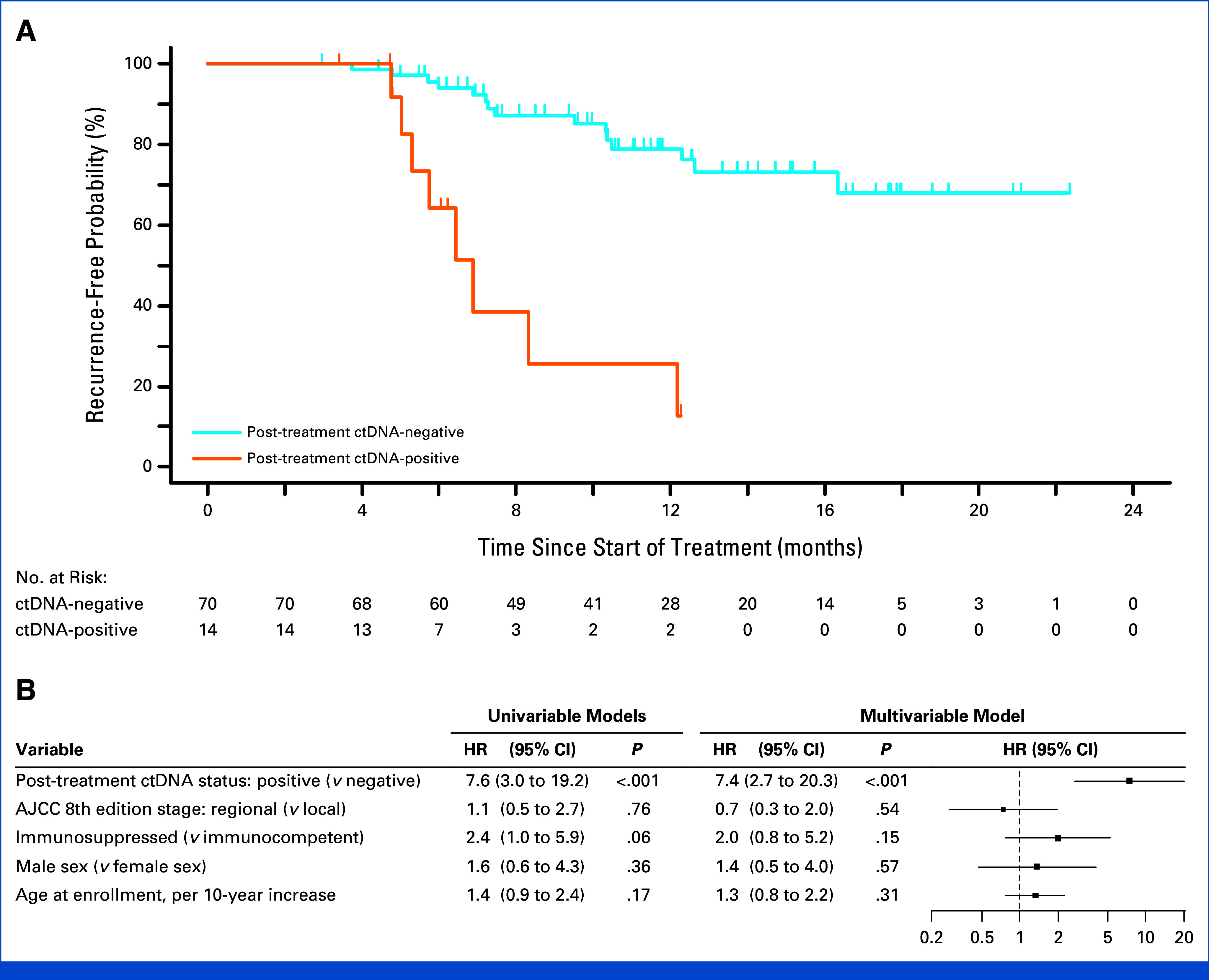
Recurrence risk stratified by initial post-treatment ctDNA status in the combined cohort. There were 84 patients with local or regional disease who underwent surgery or RT for initial treatment, became clinically negative for disease after treatment, had ctDNA measured within a 4-month post-treatment window, and had follow-up after the ctDNA test was drawn. (A) Recurrence rates were significantly higher if the first post-treatment ctDNA test was positive than if it was negative (1-year recurrence-free probability, 26% *v* 79%; *P* < .001). (B) Post-treatment ctDNA status remained significantly associated with recurrence after adjusting for stage, immunosuppression status, sex, and age (HR, 7.4 [95% CI, 2.7 to 20]; *P* < .001), and had the strongest association with outcome among these risk factors. AJCC, American Joint Committee on Cancer; ctDNA, circulating tumor DNA; HR, hazard ratio; RT, radiation therapy.

**FIG A7.**
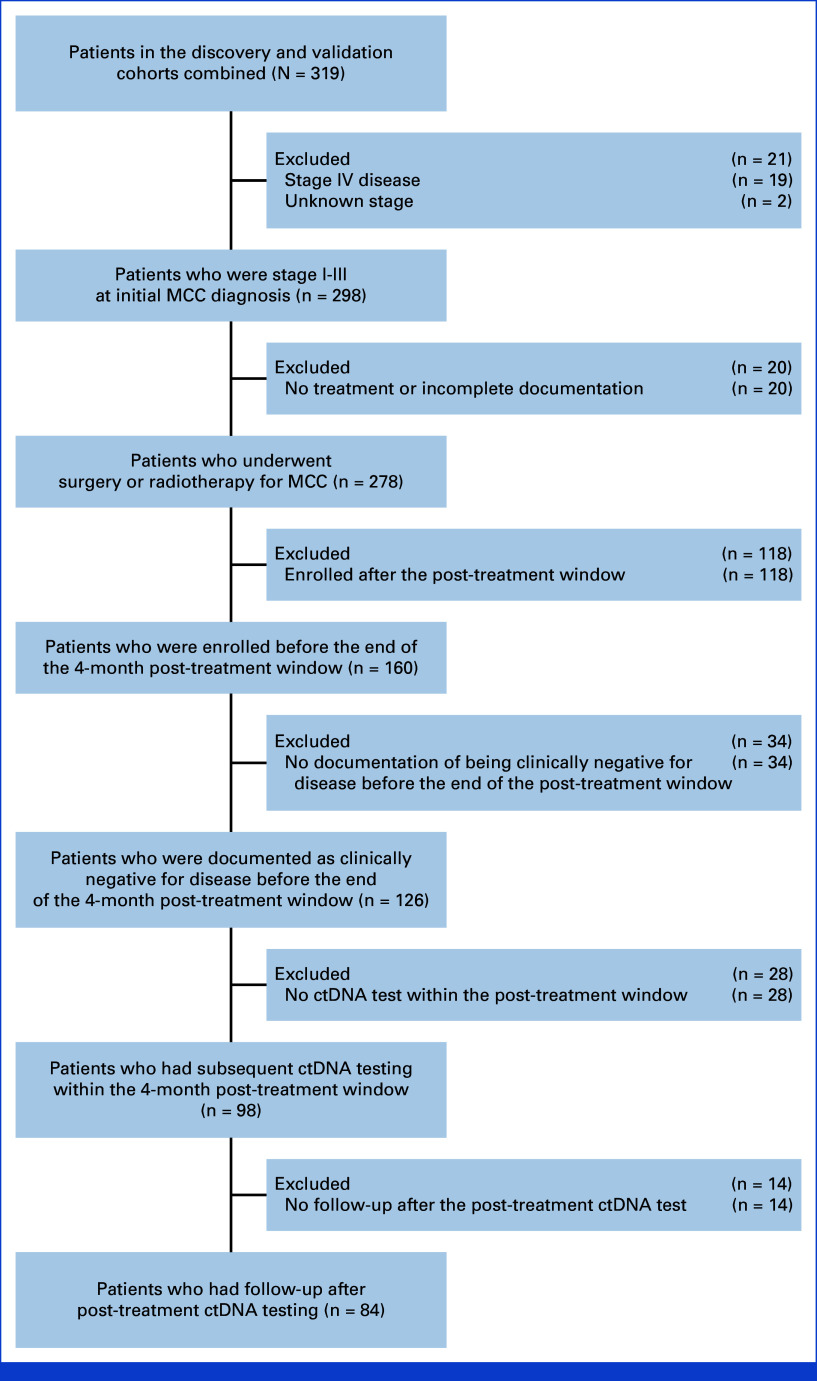
Flow diagram for the patients included in the analysis of outcomes after stratifying by post-treatment ctDNA. Patients from the discovery cohort and the validation cohort were combined in the analysis. To be eligible for the analysis, patients needed to have AJCC eighth edition stage I-III, be treated using surgery or radiation therapy, be enrolled within the 4-month post-treatment window, and be documented as clinically negative for disease during the 4-month post-treatment window. Of the 126 patients who met the preceding criteria, 28 patients were excluded because of no ctDNA testing within the 4-month post-treatment window and 14 were excluded because of lack of follow-up after their qualifying ctDNA test, leaving 84 available for analysis. AJCC, American Joint Committee on Cancer; ctDNA, circulating tumor DNA; MCC, Merkel cell carcinoma.

We then correlated post-treatment ctDNA positivity with recurrence risk in stage I-III patients. Patients who underwent a ctDNA test within 4 months after curative-intent surgery or radiation therapy were included in the analysis (flow diagram shown in Appendix Fig A7). Among these 84 patients, there were 23 recurrences and zero deaths over a median follow-up of 314 days. Compared with patients who were ctDNA-negative at the post-treatment time point (N = 70), ctDNA-positive patients (N = 14) had significantly higher recurrence rates (recurrence at 1 year: 74% *v* 21%; HR, 7.6 [95% CI, 3.0 to 19]; *P* < .001; Fig 5A). This difference in recurrence was also significant after adjusting for stage, immunosuppression status, sex, and age (HR, 7.4 [95% CI, 2.7 to 20]; *P* < .001; Fig 5B).

## DISCUSSION

In this multicenter prospective observational study of patients with stage I-IV MCC, we formally validated the utility of a tumor-informed ctDNA assay. This assay may be particularly impactful in the surveillance of patients with this highly lethal malignancy characterized by a high recurrence rate of 40% within 5 years.^1^ We demonstrated that the ctDNA assay had high sensitivity (94% in the validation cohort; Fig 2B) in detecting clinically evident disease at enrollment. Additionally, analyses of patients followed during surveillance revealed that a negative ctDNA had a very high NPV (Fig 3) and that a positive ctDNA after curative-intent treatment predicted patients with a high risk of recurrence (Fig 5).

Previous attempts to find accurate and universally effective tumor markers in MCC have fallen short. Detectable antibodies against the MCPyV are present in only 52% of patients with MCC,^5,6^ although MCPyV drives up to 80% of MCC tumors. It is a valuable biomarker in antibody-positive patients, with a PPV of 66% for clinically evident recurrence and an excellent NPV of 97% for a decreasing titer. However, its poor sensitivity in the overall MCC population limits its clinical application.

National guidelines recommend surveillance imaging for high-risk patients and as clinically indicated for others but do not specify an interval in either population.^3^ The results of our study support using ctDNA to guide imaging frequency in patients under surveillance after treatment of MCC. The high NPV of 93% (95% CI, 89 to 97) at 135 days of follow-up (Fig 3B) provides clinicians and patients with reassurance that MCC should not recur within the subsequent 3-4 months. Considering this, it may be reasonable to forgo imaging if ctDNA remains undetectable quarterly.

Conversely, ctDNA positivity during surveillance for stage I-IV patients who were clinically rendered disease-free is highly associated with recurrence (HR, 20 [95% CI, 8.3 to 50]); thus, patients with detectable ctDNA should be followed closely with physical examinations and imaging. At 12 months, the recurrence-free probability was 9% among patients with a positive ctDNA at any time during surveillance, compared with 91% for patients who remained ctDNA-negative (Fig 3A). ctDNA positivity during surveillance markedly outperforms other known factors associated with recurrence, including a history of regional or distant metastases, immunocompromised status, male sex, and age, and retains a significant association after adjusting for all the above, with a HR of 19 (95% CI, 7.1 to 51; Appendix Table A4 and Fig A5).

Additionally, in our patient cohort, ctDNA outperformed traditional recurrence risk factors in its ability to distinguish between stage I-III patients who are more likely to be cured by surgery and radiation therapy and those likely to recur (adjusted HR, 7.4 [95% CI, 2.7 to 20]; Fig 5B). Several trials (ClinicalTrials.gov identifiers: NCT02196961, NCT04291885, NCT03271372, NCT03712605) are testing the efficacy of adjuvant immune checkpoint inhibitors in patients with MCC, with results pending. A recently randomized study of adjuvant nivolumab in 179 patients with completely resected MCC showed the 24-month disease-free survival rate with nivolumab was 84% (95% CI, 76 to 90), compared with 73% (95% CI, 59 to 83) with surveillance.^28^ The HR was 0.58 (95% CI, 0.30 to 1.12; *P* = .10), but the difference was not significant. As adjuvant therapy is more justified in higher-risk patients, using ctDNA positivity after curative-intent therapy for patient selection in future studies may increase the proportion of patients who could benefit from the adjuvant therapy and increase statistical power to detect differences between treated and untreated groups.

Although our primary analysis evaluated the performance of ctDNA positivity, we also explored whether the likelihood of clinically detecting a recurrence at the time of a positive test was related to the quantitative ctDNA level. Our results show that ctDNA can identify minimal residual disease after primary treatment of MCC. The probability of clinical recurrence increased significantly with higher ctDNA levels (AUC, 0.86 [95% CI, 0.80 to 0.92]; Fig 4B; Appendix Fig A6), although the recurrence risk was still appreciable even when ctDNA levels were relatively low (17% when positive ctDNA <1 MTM/mL). This finding suggests that even low ctDNA levels should have clinical follow-up, although the ctDNA value may guide the urgency and frequency of follow-up. Further study is needed to develop more formal guidelines on interpreting the quantitative levels.

This study has several limitations. Our patient population sought treatment at tertiary care centers across the United States and thus may not be representative of all patients with MCC. There were real-world variations in primary treatment modalities and follow-up intervals for physical examinations, imaging, and ctDNA collection. Sensitivity and specificity of ctDNA were calculated on the basis of the clinical disease status assessed at the time of the first blood draw for ctDNA. This methodology does not account for new clinically evident disease subsequently found during follow-up. PPV and NPV were determined on the basis of follow-up examinations but could be affected by variation in follow-up intervals. Although our median follow-up is only 295 days, the high recurrence rate of MCC allowed for sufficient statistical power.

In summary, tumor-informed ctDNA testing is a highly accurate and prognostic biomarker for surveillance of patients with MCC, identifying low-risk patients who do not require frequent imaging, and identifying high-risk patients who require more frequent imaging. This assay can aid in early detection of recurrent or metastatic disease, although further studies with longer follow-up are needed to assess the impact on disease-specific survival. Future studies should evaluate the utility of this assay for identifying patients who are most likely to benefit from adjuvant therapy, and for monitoring tumor response to immunotherapy. Finally, as the utilization of this ctDNA assay may decrease the need for imaging follow-up in patients with undetectable levels or on systemic therapies, future studies should additionally assess the impact on the cost of care.

## APPENDIX

**TABLE A1.** Clinical Characteristics of the Patient Cohorts

Variable	Combined (N = 319)	Cohort		*P*
		Discovery (n = 167)	Validation (n = 152)	
Sex, No. (%)				.35
Male	212 (66)	115 (69)	97 (64)	
Female	107 (34)	52 (31)	55 (36)	
Age at enrollment, years, median (range)	72 (41-97)	73 (41-97)	71 (44-92)	.31
Immunosuppressed at enrollment, No. (%)	55 (17)	27 (16)	28 (18)	.66
Most recent stage at enrollment, No. (%)				.10
Local^a^	102 (32)	62 (37)	40 (26)	
Regional^b^	159 (50)	79 (47)	80 (53)	
Distant	58 (18)	26 (16)	32 (21)	
AJCC eighth edition stage at MCC diagnosis,^c^ No. (%)				.05
pI	54 (17)	38 (23)	16 (11)	
cI	28 (9)	12 (7)	16 (11)	
pII	20 (6)	14 (8)	6 (4)	
cII	26 (8)	14 (8)	12 (8)	
pIIIA	93 (29)	42 (25)	51 (34)	
pIIIB	60 (19)	27 (16)	33 (22)	
cIII	17 (5)	10 (6)	7 (5)	
IV	19 (6)	9 (5)	10 (7)	

Abbreviations: AJCC, American Joint Committee on Cancer; MCC, Merkel cell carcinoma.

^a^ Local disease defined as stages I and II.

^b^ Regional disease defined as stage III.

^c^ Stage was unavailable for two participants (one in the discovery cohort and one in the validation cohort).

**TABLE A2.** REMARK Checklist

Item to Be Reported	Description	Page No.^a^
Introduction		
1	State the marker examined, the study objectives, and any prespecified hypotheses	M1-2
Materials and methods		
Patients		
2	Describe the characteristics (eg, disease stage or comorbidities) of the study patients, including their source and inclusion and exclusion criteria	M3-4, A1-2, ST1, ST5
3	Describe treatments received and how chosen (eg, randomized or rule-based)	M3-4
Specimen characteristics		
4	Describe the type of biologic material used (including control samples) and methods of preservation and storage	M4
Assay methods		
5	Specify the assay method used and provide (or reference) a detailed protocol, including specific reagents or kits used, quality control procedures, reproducibility assessments, quantitation methods, and scoring and reporting protocols. Specify whether and how assays were performed blinded to the study end point	M4
Study design		
6	State the method of case selection, including whether prospective or retrospective and whether stratification or matching (eg, by stage of disease or age) was used. Specify the time period from which cases were taken, the end of the follow-up period, and the median follow-up time	M3-4
7	Precisely define all clinical end points examined	M3, A1, A3-7
8	List all candidate variables initially examined or considered for inclusion in models	A5
9	Give rationale for sample size; if the study was designed to detect a specified effect size, give the target power and effect size	A2-3
Statistical analysis methods		
10	Specify all statistical methods, including details of any variable selection procedures and other model-building issues, how model assumptions were verified, and how missing data were handled	M5-6, A1-8
11	Clarify how marker values were handled in the analyses; if relevant, describe methods used for cutpoint determination	M4-6, A1-8
Results		
Data		
12	Describe the flow of patients through the study, including the number of patients included in each stage of the analysis (a diagram may be helpful) and reasons for dropout. Specifically, both overall and for each subgroup extensively examined report the numbers of patients and the number of events	F1, M4-5, M7, SF6
13	Report distributions of basic demographic characteristics (at least age and sex), standard (disease-specific) prognostic variables, and tumor markers, including numbers of missing values	F4, ST1, ST5
Analysis and presentation		
14	Show the relation of the marker to standard prognostic variables	ST5
15	Present univariable analysis showing the relation between the marker and outcome, with the estimated effect (eg, hazard ratio and survival probability). Preferably provide similar analyses for all other variables being analyzed. For the effect of a tumor marker on a time-to-event outcome, a Kaplan-Meier plot is recommended	M8-9, F3, F5, ST4
16	For key multivariable analyses, report estimated effects (eg, hazard ratio) with CIs for the marker and, at least for the final model, all other variables in the model	F5, ST4
17	Among reported results, provide estimated effects with CIs from an analysis in which the marker and standard prognostic variables are included, regardless of their statistical significance	F5, ST4
18	If done, report results of further investigations, such as checking assumptions, sensitivity analyses, and internal validation	A4, A7
Discussion		
19	Interpret the results in the context of the prespecified hypotheses and other relevant studies; include a discussion of limitations of the study	M10-13
20	Discuss implications for future research and clinical value	M10-13

Abbreviation: REMARK, Reporting Recommendations for Tumor Marker Prognostic Studies.

^a^ The following prefixes were used for page numbers and numbers of figures and tables: M = main text (starting at introduction section); A = appendix; F = figure in main text; T = table in main text; SF = supplemental figure; ST = supplemental table.

**TABLE A3.** Diagnostic Performance of ctDNA at Enrollment for Merkel Cell Carcinoma Disease Status in the Combined Cohort, Stratified by Immunotherapy

Metric	On Immunotherapy at Enrollment			Not on Immunotherapy at Enrollment			*P*
	No.	Estimate, %	95% CI	No.	Estimate, %	95% CI	
Sensitivity	20/21	95	76 to 99	102/108	94	88 to 98	>.99
Specificity	22/27	81	62 to 94	139/156	89	83 to 94	.33

Abbreviation: ctDNA, circulating tumor DNA.

**TABLE A4.** Associations of ctDNA Status and Other Risk Factors With Risk of Recurrence During Surveillance

Variable	Discovery Cohort (n = 119)				Validation Cohort (n = 96)			
	Univariable Model		Multivariable Model		Univariable Model		Multivariable Model	
	HR (95% CI)	*P*	HR (95% CI)	*P*	HR (95% CI)	*P*	HR (95% CI)	*P*
ctDNA status: any positive (*v* remained negative)	6.8 (2.9 to 16.0)	<.001	7.0 (2.6 to 18.7)	<.001	20.3 (8.3 to 49.6)	<.001	19.1 (7.1 to 51.3)	<.001
Stage: regional (*v* local)	1.8 (0.7 to 4.4)	.21	1.1 (0.4 to 3.0)	.87	1.2 (0.5 to 3.2)	.69	2.4 (0.8 to 7.2)	.13
Stage: distant (*v* local)	1.1 (0.2 to 5.0)	.91	0.4 (0.0 to 4.2)	.47	1.5 (0.4 to 5.6)	.59	4.0 (0.9 to 17.4)	.06
Immunosuppressed (*v* immunocompetent)	1.9 (0.7 to 4.7)	.19	1.9 (0.7 to 5.3)	.21	2.6 (1.1 to 6.1)	.04	1.7 (0.5 to 6.0)	.41
Male sex (*v* female sex)	1.9 (0.6 to 5.6)	.26	1.5 (0.4 to 5.1)	.56	1.8 (0.7 to 4.5)	.23	1.0 (0.3 to 3.1)	.95
Age at enrollment, per 10-year increase	1.1 (0.7 to 1.9)	.62	1.0 (0.6 to 1.8)	.98	1.7 (1.1 to 2.6)	.02	1.3 (0.7 to 2.3)	.46

Abbreviations: ctDNA, circulating tumor DNA; HR, hazard ratio.

**TABLE A5.** Clinical Characteristics Stratified by ctDNA Status at the Start of the Surveillance Period

Variable	Discovery Cohort			Validation Cohort		
	ctDNA Status		*P*	ctDNA Status		*P*
	Positive (n = 17)	Negative (n = 102)		Positive (n = 16)	Negative (n = 80)	
Stage, No. (%)			.009			>.99
Local	2 (12)	48 (47)		4 (25)	19 (24)	
Regional	13 (76)	41 (40)		10 (62)	49 (61)	
Distant	2 (12)	13 (13)		2 (12)	12 (15)	
Immunosuppressed, No. (%)	3 (18)	16 (16)	.73	6 (38)	12 (15)	.07
Male sex, No. (%)	13 (76)	68 (67)	.58	11 (69)	50 (62)	.78
Age at enrollment, years, median (range)	72 (60-85)	73 (41-97)	.86	76 (57-89)	70 (45-91)	.06

Abbreviation: ctDNA, circulating tumor DNA.

**TABLE A6.** Time Between Each ctDNA Test and the Next Imaging Examination

Group	Discovery Cohort		Validation Cohort	
	No. of Tests	Time From Test to Imaging (days), Median (IQR)^a^	No. of Tests	Time From Test to Imaging (days), Median (IQR)^a^
All ctDNA tests	369	84 (42-151)	288	77 (41-107)
ctDNA-positive tests	71	55 (26-82)	66	47 (23-89)
ctDNA-negative tests	298	91 (53-174)	222	84 (46-119)

Abbreviation: ctDNA, circulating tumor DNA.

^a^ Values are median (IQR) that were estimated using the Kaplan-Meier method to account for censoring.
